# Taste-testing tarsi: Gustatory receptors for glucosinolates in cabbage butterflies

**DOI:** 10.1371/journal.pgen.1009616

**Published:** 2021-07-15

**Authors:** Noah K. Whiteman, Julianne N. Peláez

**Affiliations:** Department of Integrative Biology, University of California, Berkeley, California, United States of America; University of Kentucky, UNITED STATES

Roughly one-half of all insect species are herbivorous, and these plant-feeding insects are a critical component of all food webs on land. Yet, the terrestrial realm of our planet is verdant—from the mossy shores of Antarctica to the Amazon rainforest. Why is the world both green [1] and filled with herbivorous insects? How do we reconcile the tremendous ecological and evolutionary success of plants and the insects that eat them? There are both top-down (e.g., predators and parasites) and bottom-up (e.g., plant toxins) forces that limit herbivorous insect populations [2]. Bottom-up forces captured the imagination of Gottfried Fraenkel in 1959 [3], who proposed that the odd chemicals in plants, so-called secondary compounds, evolved not to serve the primary metabolic needs of plants, but rather as toxins to repel and harm would-be attackers. In other words, the adaptive value of these chemicals is that they are toxic to animals and shield plants from attack.

One of the pitfalls of producing a toxin is that in many cases these molecules can damage plant as well as animal cells—particularly for very broad spectrum toxins like isothiocyanates (ITCs). ITCs are produced by plants in the Brassicales (as well as in the distantly related Putranjivaceae) that give bok choy, cabbage, mustard, radish, wasabi, and watercress their spicy or peppery taste. These electrophilic, hydrophobic molecules form covalent bonds with cysteine and lysine residues and bind to DNA. ITCs are potent insecticides: Simply chopping up a fresh turnip in a jar, introducing *Drosophila melanogaster* flies, and sealing the lid results in rapid mortality [4]. ITCs have played an important practical role in modern cell and molecular biology as well. Coons and colleagues in 1942 [5] invented modern fluorescence microscopy by labeling anti-*Pneumococcus* serum from mice with a preparation of fluorescein conjugated with isocyanate. In 1958, a safer and more practical alternative to isocyanate was invented by Riggs and colleagues [6] who used an ITC instead of isocyanate. Fluorescein-ITC is now a standard reagent used to label antibodies.

Interestingly, ITCs are the end products of a chemical reaction that starts with a damaged plant cell that contains glucosinolate molecules in vacuoles. Glucosinolates are hydrophilic and nontoxic thioglucosides produced by a biosynthetic pathway evolutionarily derived from the cyanogenic glucoside pathway. When a damaged myrosin cell in the plant, in turn, releases a myrosinase (a thioglucosidase), ITCs are produced as glucosinolate hydrolysis products [7]. This is why it takes a few seconds for the tingling sensation to be perceived in our mouths as we chew on fresh tissues of Brassicales plants.

**Fig 1:**
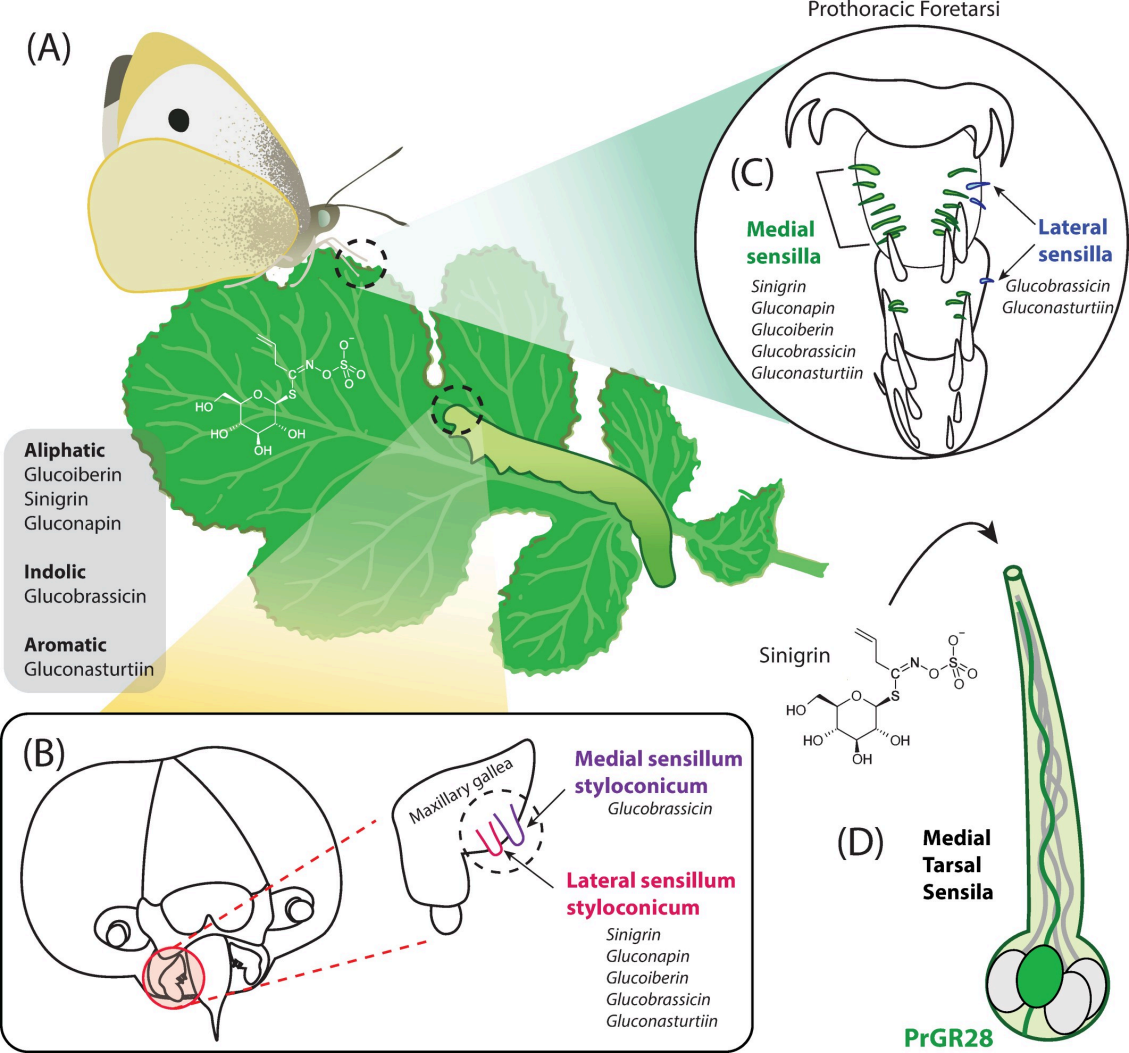
Identification of GRs underlying recognition of host plant defense compounds in the herbivorous cabbage white butterfly, *Pieris rapae*. **(A)** *P*. *rapae* are specialists on plants in the Brassicales order that produce a diverse class of defense precursor compounds, called glucosinolates, which break down upon tissue damage into toxic ITCs. Egg-laying female and leaf-feeding larvae are both influenced by the glucosinolate composition and abundance across plant tissues. Glucosinolates are classified into 3 groups: aliphatic, indolic, and aromatic; those glucosinolates tested in experiments by Yang and colleagues (this issue) are shown to the left. **(B)** Larvae taste glucosinolates with the medial (purple) and lateral (pink) sensilla styloconica found on the maxillary gallea of their mouth parts, and **(C)** adults taste them with the medial (green) and lateral (blue) sensilla on their tarsi. Through electrophysiological recordings from single sensilla, Yang and colleagues (this issue) showed that 2 sensilla types may exist in both larvae and adults: lateral sensilla styloconica in larvae and medial tarsal sensilla in adults appear broadly tuned to all glucosinolate types, whereas medial sensilla styloconica in larvae and lateral tarsal sensilla in adults are only activated by indolic and/or aromatic glucosinolates, suggesting a mechanism for distinguishing these glucosinolate classes specifically. **(D)** Using an RNAi knockdown approach of the highly expressed GR gene *PrapGR28* and a knock-in approach into the sweet neurons of *D*. *melanogaster* mutants, Yang and colleagues showed convincing evidence that PrapGr28 is highly sensitive to the glusosinolate sinigrin. GR, gustatory receptor; ITC, isothiocyanate; RNAi, RNA interference.

Because they are so limited in distribution across plants, glucosinolates are faithful signposts for herbivorous insects that specialize on these plants. One of the enduring paradoxes of herbivorous insect natural history is that most species specialize on a limited set of host plant species—usually those that produce similar toxins or toxin precursors. Indeed, in what is likely to be the first well-studied role of plant secondary compounds in mediating specialization, Verschaffelt [8] in 1910 showed that larvae of *Pieris* spp. butterflies (Fig 1) required chemicals from their mustard host plants to stimulate feeding [9] (see [10]) for a review). These molecules are glucosinolates [11] and are also required for oviposition (egg laying) [12]. ITCs are highly toxic to pierid butterflies [13], owing to the fact that these insects evolved a nitrile-specifier protein (NSP) that diverts hydrolysis of glucosinolates to less toxic nitriles insteads of ITCs [14]. Coevolution between the glucosinolate–myrosinase system of Brassicales plants and the pierid butterflies has been an exemplar system for the study of plant–insect interaction systems [15].

It is well understood that insect taste receptors (gustatory receptors or GRs) are expressed in dendrites within sensory hairs across the body, from the mouthparts to the tarsi and even the wings [16]. Further, specific GRs have been identified in swallowtail butterflies [17] that allow them to perceive a secondary compound associated with their host plants. However, in pierid butterflies, which were the very first herbivorous insects to be linked to a plant secondary compound in the context of host plant specialization, the identity of the GRs used to perceive glucosinolates was unknown until now.

In this issue, Yang and colleagues [18] report the discovery of a GR responsible for glucosinolate detection in *Pieris rapae* butterflies (Fig 1). This GR, called PrapGR28, is expressed in gustatory receptor neurons (GRNs) within the tarsi of adult females and males, as well as in larval maxillary sensilla. PrapGR28 is both highly expressed and responds selectively to sinigrin when expressed in *Xenopus* oocytes. First, the authors used electrophysiology to measure the responses of larval and adult gustatory neurons to the principle glucosinolates in cabbage, which includes sinigrin. Sinigrin is a glucosinolate found in a diversity of Brassicales plants and is already known to stimulate larval feeding and oviposition by pierids [19]. Yang and colleagues [18] found that the lateral sensilla styloconica of larvae and medial sensilla found on both adult female and male tarsi responded to sinigrin. Using a transcriptomics approach, they identified GRs with high expression in the larval and adult gustatory neurons within these tissues. Two GRs that were highly expressed in larvae and adults were identified as candidates for further study (PrapGR15 and PrapGR28), but only one, PrapGR28, was actually found to be activated by sinigrin when expressed in the *Xenopus* oocyte system.

Yang and colleagues [18] then used in vivo knock-in and knock-out studies to show that the presence or absence of PrapGR28 was sufficient to change the behavioral preference toward sinigrin. Using the toolbox of *D*. *melanogaster*, they expressed PrapGR28 in sugar-sensing GRNs using *Gr5a-GAL4* in vivo. Expressing PrapGr28 in these GRNs in fruit flies conferred sinigrin sensitivity and reduced feeding aversion compared to control flies, which suggests that despite the evolutionary distance between butterflies and flies, either PrapGR28 alone or in combination with *D*. *melanogaster* GRs can detect sinigrin. PrapGR28 is expressed in GRN cells within male and female foretarsi based on in situ hybridization. Using RNA interference (RNAi), Yang and colleagues [18] showed that the gene *PrGR28* is likely to be necessary for the gustatory response to sinigrin because in knock-down butterflies, sensitivity to sinigrin was reduced.

The identification of GRs responsible for detecting important host plant compounds presents a major advance toward understanding the neuroethology and sensory ecology of herbivorous insects, where functional characterization in non-drosophilids has only recently begun to mount [20–22]. Identifying receptors able to detect token stimuli paves the way for understanding how herbivorous insects identify appropriate, high-quality feeding and egg-laying locations. Specifically, we can begin to understand which host plant compounds are detected by which receptors, and within a “labeled line” neural system, how the expression patterns of receptors within distinct gustatory neurons can lead to differential perception of diverse host plant compounds. Research on *P*. *rapae* has shown that indolic glucosinolates are the strongest oviposition stimulants, in comparison to the more toxic aliphatic glucosinolates, which may be more harmful to developing larvae. While sinigrin (aliphatic) can be detected by PrapGR28 without the aid of any other GRs, it remains to be determined which GRs detect the other glucosinolates that are attractive to *P*. *rapae* and whether and how these GRs form multimers with one another. For instance, RNAi knockdown of *PrapGR28* in *P*. *rapae* caused reduced sensitivity to gluconapin, but expression of only PrapGr28 in *D*. *melanogaster* sweet GRNs was not sufficient to elicit a response to gluconapin.

Based on the electrophysiological recordings from larval and adult tarsal sensilla, Yang and colleagues [18] present the intriguing finding that, at least from testing five glucosinolates, there exist two types of sensilla in both larvae and adults—one broadly tuned (responds to all five glucosinolates) and one narrowly (only responds to the indolic and aromatic glucosinolates) (Fig 1). More glucosinolates will need to be tested—only one indolic and aromatic glucosinolate each were tested—to further investigate this hypothesis. But as Yang and colleagues [18] suggest, if true, this would be one way that herbivorous insects could discriminate more attractive, less toxic glucosinolates (indolic/aromatic) from the overall level of glucosinolates present in plant tissues. It will be important to identify receptors tuned to these stronger feeding and oviposition stimulants and parse the localization patterns of these receptors among GRNs and sensilla types. Much still remains to be deciphered about the role of and molecular mechanisms by which GRs operate to elicit aversion or attraction toward their host plants. But through elegant studies, like that of Yang and colleagues [18], where electrophysiological, transcriptomic, and functional genetic approaches are combined, we are coming ever closer to unraveling the taste systems of the earth’s most dominant animal guild: voracious herbivores.
